# Lipoprotein(a) Response to Dietary Saturated Fat Reduction: Relationship to Apolipoprotein(a) Size Polymorphism in African Americans

**DOI:** 10.3390/nu17030426

**Published:** 2025-01-24

**Authors:** Hayley G. Law, Munkhtuya Myagmarsuren, Heejung Bang, Wei Zhang, Michael Lefevre, Lars Berglund, Byambaa Enkhmaa

**Affiliations:** 1 Department of Internal Medicine, School of Medicine, University of California Davis, One Shields Avenue, Davis, CA 95616, USA; haglaw@ucdavis.edu (H.G.L.); mmyagmarsuren@ucdavis.edu (M.M.); wzhang@ucdavis.edu (W.Z.); lberglund@ucdavis.edu (L.B.); 2 Department of Public Health Sciences, School of Medicine, University of California Davis, One Shields Avenue, Davis, CA 95616, USA; hbang@ucdavis.edu; 3 Department of Nutrition, Utah State University, Old Main Hill, Logan, UT 84322, USA; michael.lefevre@usu.edu

**Keywords:** DASH diet, Lp(a), saturated fatty acid, apo(a) size, apo(a) phenotype, African Americans

## Abstract

Background/Objectives: An elevated lipoprotein(a) [Lp(a)] level, which is a prevalent cardiovascular risk factor, is genetically determined by a size polymorphism of its apolipoprotein(a) [apo(a)] component. Despite its genetic control, Lp(a) level increases in response to dietary saturated fat (SFA) reduction. We tested the roles of apo(a) size and characteristics in modulating Lp(a) response to SFA reduction. Methods: We assessed apo(a) characteristics in 165 African Americans experiencing a 24% Lp(a) increase resulting from SFA reduction [16% at an average American Diet diet (AAD) to 6% at a DASH-type diet]. Apo(a) effects were tested based on the following factors: (1) the presence of a small atherogenic size (≤22 kringles), (2) phenotype (single or two isoforms), (3) isoform dominance, and (4) tertiles of combined kringle sizes. Results: There were no significant differences in Lp(a) increases between carriers vs. non-carriers of a small apo(a), between those with a single vs. two expressed isoforms, or in those with differing isoform dominance patterns (*p* > 0.05 for all). The extent of Lp(a) increase differed across increasing tertiles of combined kringle sizes (*p* = 0.006 for trend). In a multivariate model, the AAD Lp(a) level was a significant predictor of Lp(a) changes (*p* < 0.05). Relative increases in the allele-specific apo(a) level—an Lp(a) level associated with a defined apo(a) size—were similar across the apo(a) size spectrum. Conclusions: Reducing dietary SFA intake results in a 24% increase in Lp(a) level in African Americans across apo(a) sizes. Individuals with smaller apo(a) sizes reached an elevated Lp(a) level post-intervention compared to those with larger sizes, in some cases resulting in cardiovascular risk reclassification.

## 1. Introduction

Lipoprotein(a) [Lp(a)] is an atherogenic lipoprotein with an extensive size variability due to a polymorphism of the kringle (K) IV component of apolipoprotein(a) [apo(a)] [1,2,3,4]. An inverse relationship between the number of kringle repeats and Lp(a) level results in smaller apo(a) isoforms, correlating with higher Lp(a) plasma levels and a higher risk of cardiovascular disease (CVD) compared to larger apo(a) isoforms [5,6,7,8]. Lp(a) levels are elevated in African Americans compared with other population groups, and this difference cannot be fully explained by genetic factors [9,10,11,12,13].

While genetic factors are important regulators of Lp(a) levels, evidence suggests that non-genetic factors, including diet, also play a role. Most available evidence from well-designed dietary studies suggests that replacing saturated fatty acids (SFAs) with other macronutrients—such as monounsaturated fats or complex carbohydrates—increases Lp(a) levels while decreasing LDL-C concentration [14,15,16]. Notably, the reducing effect of dietary SFAs on Lp(a) levels contrasts with the well-known atherogenic and cholesterol-raising properties of SFAs [16]. We recently demonstrated that Lp(a) levels increased by 24% in response to a 5-week intervention replacing dietary SFAs with complex carbohydrates among a large number of African Americans enrolled in GET-READI (the Gene-Environment Trial on Response in African Americans to Dietary Intervention), a randomized, controlled, cross-over metabolic feeding trial [17]. In contrast, the LDL-C concentration decreased by 10% in response to the same intervention [17].

Although growing evidence now supports the potentially detrimental opposite change in Lp(a) level to LDL-C level during healthy diet interventions, it remains unknown whether the Lp(a)-increasing effect of dietary SFAs reduction is influenced by apo(a) size and its phenotypic characteristics. To address this knowledge gap, we investigated the impact of apo(a) sizes and phenotypes on individual responses in Lp(a) levels as well as in allele-specific apo(a) levels in the GET-READI participants. Additionally, we tested the relationship between apo(a) characteristics and LDL-C reduction to gain further insights into the opposite responses of Lp(a) and LDL-C to dietary SFAs modulation.

## 2. Materials and Methods

### 2.1. Study Population

Details of the GET-READI trial, including inclusion and exclusion criteria, have been described previously [17]. Briefly, 166 participants were recruited by the Pennington Biomedical Research Center from the greater Baton Rouge, Louisiana area. African American full sibling pairs with a parent available for genetic testing or groups of ≥3 African American full siblings, regardless of parents’ availability for testing, were recruited into the GET-READI study. At least one sibling was required to have an LDL-C above the 50th percentile when adjusted for age, race, and sex. The exclusion based on age (<18 and >45 years) was chosen to give the greatest range possible while avoiding the complications of maturation on the low end and of aging on the high end. Exclusions based on duplicate screening laboratory values included (1) LDL-C ≥ 200 mg/dL; (2) triglycerides ≥ 500 mg/dL; and (3) blood pressure that was 160 mmHg systolic or 95 mmHg diastolic. These upper limits were set to exclude individuals who would be better served by following a more severe diet plan or being considered for drug therapy. Additional key exclusion criteria for the participating offspring included (1) documented presence of atherosclerotic disease; (2) diabetes mellitus; (3) taking antihypertensive medication; (4) renal, hepatic, endocrine, gastrointestinal, or other systemic disease; (5) BMI ≥ 40; (6) for women, pregnancy, breast feeding, or being < 6 months postpartum; (7) history of drug or alcohol abuse; (8) history of depression or mental illness requiring treatment or medication within the last 6 months; and (9) chronic use of over-the-counter medication which would interfere with study endpoints, including nonsteroidal anti-inflammatory drugs, laxatives, and antacids. The study followed the Declaration of Helsinki Principles and was approved by the Institutional Review Boards of the Pennington Biomedical Research Center (IRB #PBRC22031 approved on 8 January 2003) and University of California Davis (IRB #UCD1789275 approved on 23 July 2021). Written informed consent was obtained from all participants prior to the commencement of the study.

### 2.2. Intervention Diets

Participants were fed two diets: an Average American Diet (AAD) containing 16% kcal from SFAs, and a diet similar to the Dietary Approaches to Stop Hypertension (DASH-type) diet with 6% kcal from SFAs [17]. Intervention periods lasted 5 weeks each, with a 1-week break between the diet periods. The order of interventions was randomized and only study foods were permitted during the feeding phases of the trial. All personnel involved in determining outcome variables were blinded regarding the test diets. Calorie levels were held at a weight maintenance level throughout both intervention periods. Potentially confounding nutrients, such as dietary cholesterol, were held constant to minimize unintended differential dietary effects. Compliance checks included meal tray inspections, participant diet diaries, and measurements of participants’ urinary potassium, sodium, and magnesium levels [17]. Specifically, the mean (SD) urinary potassium (65 ± 30 vs. 36 ± 14 mmol/L) and magnesium (9.4 ± 4.6 vs. 6.8 ± 3.5 mmol/L) levels were higher in participants following the DASH-type diet vs. the AAD. In accordance with the study design, which aimed to hold sodium levels constant across diets at palatable levels, the mean urinary sodium levels were similar at the end of the AAD vs. at the end of the DASH-type diet (124 ± 53 vs. 119 ± 55 mmol/L). Additionally, as previously reported [17], total cholesterol (169 ± 31 vs. 186 ± 32 mg/dL, *p* < 0.0001), LDL cholesterol (103 ± 26 vs. 115 ± 28 mg/dL, *p* < 0.0001) and glucose (92 ± 10 vs. 93 ± 11 mg/dL, *p* = 0.045) concentrations were significantly lower following the DASH-type diet compared with the AAD. Changes in other measured biomarkers, including inflammatory biomarkers, are shown in Table S1.

### 2.3. Determinations of Lp(a) and Allele-Specific apo(a) Levels and Apo(a) Characteristics

Concentrations of Lp(a) (and LDL-C) were measured in fasting plasma samples with a rate nephelometry assay and a Beckman array system, respectively, at the Pennington Biomedical Research Center’s clinical chemistry laboratory (Baton Rouge, LA, USA) [17]. The averages of replicate measurements taken at the end of the AAD or DASH-type diet were used for analyses. Apo(a) isoform sizes were determined using Western blotting following a protocol based on the work of Kamboh et al. [18]. Briefly, plasma samples were loaded onto SDS-agarose gels for protein separation followed by immunoblotting. Apo(a) isoform sizes were determined using Lp(a) standards with known apo(a) isoform sizes (TechnoClone GmbH, Vienna, Austria). One participant had no detectable apo(a) band(s) following repeated Western blotting and was excluded from further analysis. Thus, the present analysis is based on data from 165 participants. Participants were identified as carriers of a small apo(a) isoform size if at least one apo(a) isoform size was ≤22 K. The apo(a) phenotype was based on the number of expressed protein bands and defined as single or two isoforms. In additional analyses of data from participants with two expressed apo(a) isoforms, the sum of the two apo(a) sizes was used to stratify participants into tertiles of combined kringle sizes. Dominance patterns of apo(a) isoforms were determined as previously described and defined as co-dominating, smaller-dominating, or larger-dominating [11,19]. We also determined allele-specific apo(a) level, i.e., an Lp(a) level associated with a defined apo(a) size, based on the expression level of apo(a) protein bands on Western blot in each participant, as described in our previous studies [11,19].

### 2.4. Statistics

We used standard descriptive statistics to summarize variables, including participants’ characteristics, with mean ± standard deviation (SD) or median (interquartile range [IQR]) if skewness was high for continuous variables, and frequency and percentage for categorical variables. When we made inferences about the mean, we reported the SD. For variable changes from the end of the AAD to the end of the DASH-type diet, we derived changes in unit values and percent changes per the following calculations: (DASH-type value − AAD value), and (DASH-type value − AAD value)/AAD value × 100%, respectively [17]. The test variables were natural log-transformed prior to analysis if the data skew was high. After the equality of variance was checked, unpaired *t*-tests were used to test for significant changes between group pairs. One-factor ANOVA was used to test for the significance of changes between more than two groups. For categorical variables, we used Fisher’s exact test to compare the proportion in comparison groups. Box plots display the distribution of changes in Lp(a) between diets among full samples as well as in prespecified subgroups. Furthermore, regression analysis fit via mixed-effects models with a random intercept for each family were used to test for relationships between Lp(a) changes and differences in apo(a) and demographic characteristics in all participants. As our study represented an exploratory/secondary analysis, no adjustments were made for multiplicity in statistical comparisons. We used SAS version 9.4 for data analyses.

## 3. Results

### 3.1. Participant Characteristics

**Figure 1:**
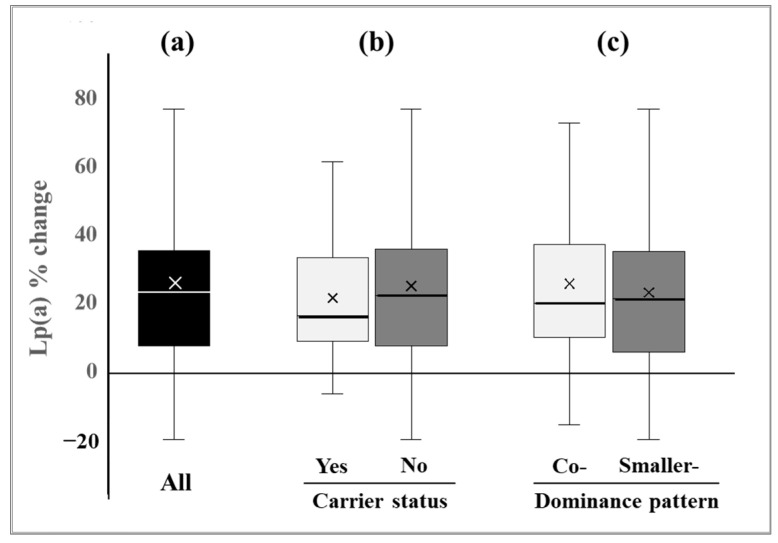
Changes in Lp(a) levels from the AAD to the DASH-type diet in all participants and by carrier status of a small-size apo(a) or apo(a) dominance pattern. Box plots represent distributions of percent (%) changes in Lp(a) levels from the Average American Diet (AAD) to Dietary Approaches to Stop Hypertension (DASH)-type diet in (**a**) all participants, (**b**) carriers (n = 26) vs. non-carriers (n = 139) of a small (≤22 kringles) size apolipoprotein(a), and (**c**) participants with co-dominating (n = 73) vs. smaller-dominating (n = 39) apolipoprotein(a) dominance patterns. Straight lines inside the boxes represent the median, with outer edges showing the 25th and 75th percentiles. The “x” symbols and whiskers mark the mean and maximum and minimum values, respectively.

The characteristics of all 166 GET-READI participants have been previously described for the entire cohort and for men and women separately [17]. Briefly, the mean (± SD) age was 35 ± 11 years, ~70% of participants were women, the average body mass index (BMI) was 28 ± 5 kg/m^2^, and the average diastolic and systolic blood pressures were 75 ± 7 mmHg and 118 ± 10 mmHg, respectively. Lp(a) levels were higher following the DASH-type diet compared with the AAD [median (IQR): 58 (29–94) mg/dL vs. 44 (22–80) mg/dL], resulting in a significant increase (mean ± SD) of 11 ± 11 mg/dL, corresponding to a 24 ± 25% relative increase (*p* < 0.0001) [17] (Table 1, Figure 1a).

The apo(a) analysis was based on data representing all GET-READI participants with detectable apo(a) bands (n = 165). The median (IQR) sizes for the larger and smaller apo(a) isoforms were 32 K (29–34 K) and 27 K (24–29 K), respectively (Table 1). Twenty-six (16%) participants had at least one small apo(a) isoform, defined as 22 K. The prevalence of phenotypes with a single vs. two expressed apo(a) isoforms was 29% (n = 48) and 71% (n = 117), respectively. Among participants with two expressed apo(a) isoforms, a co-dominating pattern regarding isoform size dominance was most common (63%), followed by a pattern with smaller size dominating (33%). Only five participants (4%) had a dominating larger isoform size (Table 1).

### 3.2. Lp(a) Response by Carrier Status of a Small Size Apo(a), Apo(a) Phenotype and Dominance Pattern

As expected, Lp(a) levels at the end of both the AAD and the DASH-type diet periods were significantly higher in carriers vs. non-carriers of a small (≤22 K) apo(a) [median (IQR)]: 100 (47–120) mg/dL vs. 38 (21–68) mg/dL (*p* < 0.0001) and 111 (53–129) mg/dL vs. 52 (26–85) mg/dL (*p* < 0.0001), respectively (Table 2). The mean level change in Lp(a) between the AAD and the DASH-type diet did not differ significantly between carriers and non-carriers of small-size apo(a): 15 ± 14 mg/dL vs. 11 ± 11 mg/dL, respectively (*p* = 0.114). The corresponding relative changes in Lp(a) levels were 21 ± 20% in carriers vs. 25 ± 26% in non-carriers of a small-size apo(a) (*p* = 0.518) (Table 2, Figure 1b).

Lp(a) levels at the end of the AAD and DASH-type diet periods were significantly higher among participants with two expressed vs. a single expressed isoform [median (IQR)]: 52 (30–88) mg/dL vs. 29 (15–55) mg/dL (*p* = 0.0003) and 65 (37–102) mg/dL vs. 34 (17–67) mg/dL (*p* = 0.0005), respectively. However, the changes in Lp(a) levels from the AAD to the DASH-type diet did not differ significantly between the two groups (mean ± SD): 12 ± 11 mg/dL vs. 9 ± 11 mg/dL for individuals with two isoforms vs. a single isoform, respectively (*p* = 0.153), resulting in corresponding relative changes of 24 ± 24% and 26 ± 29% (*p* = 0.662). When the 117 participants with two expressed apo(a) isoforms were stratified into groups of co-dominating, smaller-dominating, or larger-dominating apo(a) isoform patterns, no significant differences in Lp(a) level or changes were found (Table S2). As only five participants (4%) had a larger-dominating pattern, the Lp(a) response for the co-dominating (n = 73) and smaller-dominating (n = 39) groups is shown in Figure 1c.

### 3.3. Lp(a) Response by Tertiles of Combined apo(a) Kringle Sizes in Participants with Two Expressed Apo(a) Isoforms

We explored the impact of the combined apo(a) sizes on Lp(a) changes in participants with two expressed isoforms (n = 117), dividing the group into tertiles. During both the AAD and DASH-type diet interventions, median Lp(a) levels gradually decreased as the combined kringle size increased (Table 3). Following the DASH-type diet, the Lp(a) levels across apo(a) size tertiles were 111 (88–129) mg/dL, 64 (41–91) mg/dL, and 39 (20–62) mg/dL for tertiles 1, 2, and 3, respectively (*p* < 0.0001). The extent of increase in the Lp(a) level showed a significant gradual decrease across tertiles 1–3: 15 ± 14 mg/dL, 14 ± 10 mg/dL, and 8 ± 9 mg/dL, *p* = 0.024 for differences between tertiles, and *p* = 0.006 for trend (Table 3). Furthermore, we determined the distributions of apo(a) isoform dominance patterns and the carrier status of a small-size apo(a) across tertiles. While a co-dominating apo(a) isoform pattern was more common in tertiles 1 and 2 (68% and 72%, respectively), a smaller-dominating apo(a) pattern was more common in tertile 3 (52%). Over half of the individuals had at least one small-size (≤22 K) apo(a) in tertile 1 (56%), but only three (7%) were carriers in tertile 2, and no carriers were seen in tertile 3 (Table 3).

### 3.4. Determinants of Changes in Lp(a) Levels from the AAD to the DASH-Type Diet in All Participants

We analyzed all participants using a regression model fit via mixed-effects models with a random intercept for family groups (as participants were recruited as sibling pairs). Changes in Lp(a) levels between the AAD and the DASH-type diet were significantly associated with the AAD Lp(a) level either before or after adjusting for family status (before adjustment β = 0.113, *p* < 0.001; after adjustment β = 0.098, *p* < 0.001 for level; before adjustment β = −0.126, *p* = 0.014; after adjustment β = −0.1440, *p* = 0.032 for relative change) (Table 4). In addition, the size of the smaller apo(a) isoform was significantly associated with the Lp(a) level change before adjustment (β = −0.832, *p* < 0.001), but not after adjustment for covariates (β = −0.354, *p* = 0.404) (Table 4). There were no relationships between Lp(a) changes and anthropometric characteristics such as age or sex.

### 3.5. Changes in Allele-Specific Apo(a) Levels Across the Apo(a) Size Spectrum in All Participants

**Figure 2:**
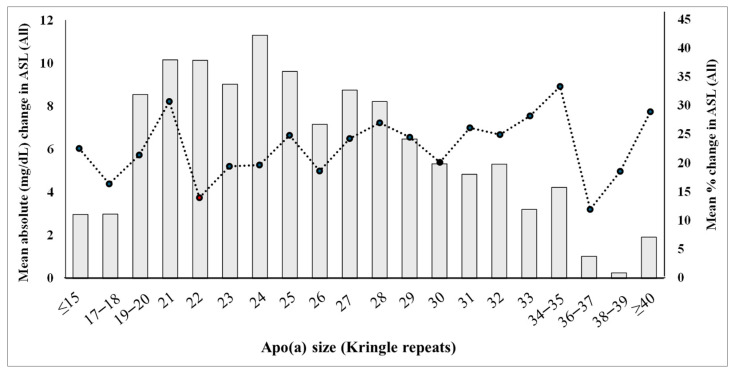
Changes in allele-specific apo(a) levels from the AAD to the DASH-type diet across the apo(a) size spectrum in all participants. The bar graphs represent the mean absolute (mg/dL) changes in allele-specific apo(a) levels from the Average American Diet (AAD) to the Dietary Approaches to Stop Hypertension (DASH)-type diet across the apo(a) size spectrum (12−45 kringle repeats) in all participants (n = 165) (scales are on the left). The dotted line graphs represent the corresponding mean relative (%) changes in allele-specific apo(a) levels (scales are on the right). Allele-specific apo(a) levels were determined by apportioning Lp(a) plasma levels to defined apo(a) sizes in a given individual based on the expression level of apo(a) protein bands, as described previously [11,19].

As the AAD Lp(a) level significantly predicted Lp(a)’s response to dietary SFAs reduction, we assessed changes in allele-specific apo(a) levels in all participants across the apo(a) size spectrum. As seen in Figure 2, although the smallest apo(a) sizes (≤18 K) had a lower allele-specific apo(a) level, the trend after that was clear, showing a gradual decrease in allele-specific apo(a) levels over the increasing number of Kringle repeats. In contrast, the relative (percentage) increase in allele-specific apo(a) levels across the apo(a) size spectrum remained relatively constant (Figure 2), arguing against any selective increases in the relative proportion of apo(a) sizes within any individual. These findings indicate that a higher allele-specific level in AAD reached a higher absolute level than a lower allele-specific level in AAD following the DASH-type diet intervention.

### 3.6. LDL-C Response and Apo(a) Characteristics

Next, we explored the relationship between changes in LDL-C concentration and apo(a) size and characteristics. In all participants, the mean LDL-C concentration was lower in those following the DASH-type diet compared with the AAD (103 ± 26 mg/dL vs. 115 ± 28 mg/dL), resulting in a significant decrease of −12 ± 15 mg/dL (corresponding to a percent decrease of −10 ± 12%) (*p* < 0.0001 for both level and percent changes) [17]. The extent of LDL-C decrease did not significantly differ between carriers vs. non-carriers of a small-size apo(a) (Table S3), by apo(a) isoform dominance patterns (Table S4), or across tertiles of combined apo(a) kringle sizes (Table S5).

## 4. Discussion

To our knowledge, this is the first study to examine the role of the apo(a) polymorphism—the major genetic regulator of Lp(a)—in dietary modulation of Lp(a) levels based on a randomized, controlled, metabolic feeding trial among a large number of African Americans. The GET-READI study design enabled the unique assessment of intra-individual Lp(a) response to dietary SFAs reduction and replacement by complex carbohydrates [17]. The assessment of apo(a) sizes in each individual allowed us to determine the impact of the dietary regimen throughout a range of allele-specific Lp(a) levels. Despite our finding that the Lp(a) level at the end of the AAD (baseline) was a key determinant of the Lp(a) response to a reduction in dietary SFAs intake, it is also important to emphasize that the relative increase in Lp(a) levels was similar across the apo(a) size range. Thus, individuals with higher allele-specific apo(a) levels in AAD reached higher levels during the DASH-type diet, potentially resulting in an increased CVD risk. These findings suggest that while apo(a) properties may not predict changes, there is nevertheless an impact, as the absolute level reached (and hence cardiovascular risk) differs. Another key novel finding was that apo(a) size variability and related characteristics did not modulate the LDL-C-decreasing effect of reducing dietary SFAs intake. Overall, our findings provide insights into how genetic and dietary factors may interact to influence an individual’s Lp(a) level and global cardiovascular risk, which is a significant knowledge gap [20].

In the present study, the Lp(a) response was similar for carriers and non-carriers of an atherogenic, small-size apo(a). Both groups experienced a >20% increase in their Lp(a) levels resulting from the DASH-type diet. Due to the continuous association between Lp(a) levels and cardiovascular outcomes, any increase in Lp(a) level might confer additional CVD risk [21]. In a recent consensus statement, an Lp(a) level >50 mg/dL has been “ruled-in” as a level at which Lp(a) may contribute significantly to CVD risk, regardless of the presence of other CVD risk factors [21,22]. Thus, Lp(a) increases in carriers of a small-size apo(a) may result in a relatively greater impact on CVD risk given their median AAD Lp(a) level (100 mg/dL), which far exceeds the 50 mg/dL associated with CVD risk [21,22]. However, while levels were lower, Lp(a) levels did also increase significantly following adherence the DASH-type diet among non-carriers. An Lp(a) range of 30–50 mg/dL was defined in the same consensus statement as the “gray zone” for Lp(a)-attributable CVD risk [21,22]. In the present study, 57 individuals had an AAD Lp(a) level of ≤30 mg/dL, and 13 of these individuals (23%) moved up to the gray zone (30–50 mg/dL category), while two individuals (4%) surpassed this zone with Lp(a) levels following the DASH-type diet of >50 mg/dL (“ruled-in”). Notably, among the 36 individuals with an Lp(a) level of 30–50 mg/dL at AAD, 18 individuals (50%) shifted their risk-associated category from the gray zone to the ruled-in zone (>50 mg/dL) at the end of the DASH-type diet. An overview of this Lp(a)-attributable risk category shift due to the DASH-type diet intervention is shown in Figure S1. As risk estimation is based on concentrations of circulating Lp(a), these shifts in risk categories due to dietary changes present a key area of interest and emphasize the importance of a precision nutrition approach to CVD prevention. An estimated 1.4 billion people worldwide have Lp(a) levels > 50 mg/dL [23]. Thus, the observed increase in Lp(a) levels for both smaller and larger apo(a) sizes following a diet consistent with recommended population-level guidelines could modify CVD risk for numerous individuals. Our findings indicate that individuals with high or very high Lp(a) levels might be subject to increased CVD risk by consuming these diets. The high population prevalence (~25%) of elevated Lp(a) signifies the public health importance of addressing knowledge gaps in our understanding of the dietary modulation of Lp(a) and the key role of personalized nutrition. Our findings reinforce the importance of including the Lp(a) level in a comprehensive assessment of an individual’s unique CVD risk profile and in implementing personalized preventive and therapeutic measures.

Though there are limited data examining the relationship of the apo(a) size polymorphism with non-genetic regulators of Lp(a) levels, one notable exception is non-nephrotic chronic kidney disease (CKD). Elevations in the Lp(a) level occur in CKD patients vs. controls in a size-specific manner, i.e., larger, but not smaller, apo(a) isoforms are associated with 2- to 4-fold higher Lp(a) levels in CKD patients compared to isoform-matched healthy controls [24,25]. In the current study, we observed an increase in the Lp(a) level between the two intervention diets regardless of apo(a) size. While we found a greater response for individuals with certain apo(a) characteristics, our results suggest that reductions in dietary SFAs may increase an individual’s Lp(a)-attributable CVD risk, regardless of their genetically determined apo(a) size. However, as discussed above, while the relative change in Lp(a) levels was similar across apo(a) sizes, the apo(a) size impacted the absolute change. Collectively, our results and those for CKD patients indicate differing mechanisms for non-genetic metabolic regulation of Lp(a) levels, highlighting the need for a better understanding of underlying mechanisms.

An opposite response of Lp(a) and LDL-C to dietary SFAs reduction has been consistently shown [14,15,16], though the exact relationship and risk balance between the two CVD risk factors remains incompletely understood. The current findings suggest that lowering of LDL-C through SFAs reduction is largely independent of the apo(a) size polymorphism. These results indicate differing metabolic mechanisms controlling LDL-C and Lp(a) levels during dietary SFAs reduction and emphasize the need to further explore the processes driving the inverse responses of these two atherogenic lipoproteins. In this context, it is important to note the ongoing clinical endeavors to test the Lp(a) hypothesis, i.e., whether lowering high concentrations of Lp(a) would lead to reductions in cardiovascular outcomes. Over the past decade, several Lp(a)-lowering drugs with potent effects [up to a 98% maximum reduction in Lp(a)] have been developed, and some of these drugs are already being tested in phase 3 cardiovascular endpoint trials [26,27,28,29,30,31]. These Lp(a)-lowering drugs lower Lp(a) synthesis through gene silencing or by preventing apo(a) from binding to LDL particles [31].

The potential relationship between Lp(a) level, apo(a) size, and diet has been explored in a few observational cohort studies. Marcovina et al., reported that participants eating a fish-based diet had significantly lower levels of Lp(a) compared to participants eating a vegetarian diet taking apo(a) sizes into account [32]. This study found no significant effect of apo(a) size on differences in Lp(a) level when examining diets’ effects. Matveyenko et al. found a significant negative relationship between dietary SFAs intake and Lp(a) level in a free-living, diverse cohort controlling for self-reported race/ethnicity and apo(a) isoform size [33]. While these reports are in line with our findings, our current interventional study moved beyond the observational level, as we uniquely controlled for diet composition in order to limit any impact of potentially confounding nutrients, which were held constant, and SFAs intake was the major difference between the two diets. A recent systematic review concluded that compared to higher-SFA diets, lower-SFA diets modestly increase Lp(a) levels among individuals without atherosclerotic CVD [34]. This effect appeared to be driven by replacement of SFAs with carbohydrates or trans fatty acids. The findings suggest a specific effect of reducing dietary SFAs on the Lp(a) level, where the degree of change might be determined by other macronutrient choices. It is notable that the Lp(a) level is primarily determined by its synthesis rate [35]; however, the mechanism behind the effect of SFAs remains to be determined. In this context, it is interesting to note the Lp(a) increase across the apo(a) size spectrum following dietary SFAs reduction. As shown previously, large apo(a) isoforms are more susceptible than smaller isoforms to proteosomal degradation [35,36], resulting in higher Lp(a) levels in carriers of small apo(a) isoforms, as also observed in the present study. While the mean changes in Lp(a) levels were higher for smaller vs. larger apo(a) sizes, it is important to note that the corresponding relative changes were similar across the apo(a) size spectrum.

Our study has some strengths and limitations. While we studied a large number of African Americans using a highly informative method for apo(a) assessment enabling detection of two individual apo(a) isoforms, additional studies are needed to determine any role of other genetic variants in modulating the Lp(a) response to dietary interventions. This is notable in the context of the potential genetic heterogeneity of African Americans, whose genetic markers could be more polymorphic compared to others. Despite employing one of the largest randomized controlled dietary trials, we recognize the potential limitations in the statistical power of subgroup analyses. Although studies in other populations have shown a similar direction of Lp(a) response to dietary SFAs reduction [14,15,37], the generalizability of the current apo(a) findings to other population groups require further investigations. Furthermore, the allele-specific apo(a) level was based on the data collected at a single time point (either in AAD or in DASH-type diet) for a given individual. While this approach may require confirmation in dietary studies, we have shown equal or no changes from baseline in the relative apo(a) expression in the majority of participants receiving a drug treatment that substantially reduced Lp(a) levels [19]. Our study, which was designed to assess differences in Lp(a) levels between the AAD and the DASH-type diet at the end of each diet period, limited our ability to study any dynamic changes in Lp(a) within each diet period. Finally, the relative risk balance of the opposite changes in Lp(a) and LDL-C concentrations following dietary SFAs reduction needs to be comprehensively mapped, perhaps in the context of an individual’s unique genetic architecture and cardiovascular risk, as in precision medicine.

## 5. Conclusions

In conclusion, reducing dietary saturated fat intake resulted in a significant 24% increase in Lp(a) level in African Americans across apo(a) sizes. Furthermore, individuals with smaller apo(a) sizes reached an elevated Lp(a) level post-intervention compared to those with larger sizes, in some cases resulting in a CVD risk reclassification. This underscores the clinical implication of the universal recommendation to reduce dietary SFAs intake and the need for more personalized nutrition approaches. Additionally, apo(a) size and characteristics did not impact the degree of LDL-C lowering during a reduction in dietary saturated fat intake. While the findings require confirmation in other diverse cohorts, they contribute to advancing our understanding of the dietary modulation of a highly heritable prevalent risk factor for CVD.

## Figures and Tables

**Table 1:** Lipoprotein(a) and apolipoprotein(a) characteristics in all participants.

Variables	All
Lp(a) level	
Average American Diet (mg/dL)	44 (22–80)
DASH-type diet (mg/dL)	58 (29–94)
Unit (mg/dL) change	11 + 11 ^1^
Percent (%) change	24 + 25 ^1^
Apo(a) isoform size (kringle repeats) ^2^	
Larger isoform	32 (29–34)
Smaller isoform	27 (24–29)
Carrier status of a small (<22 kringles) apo(a) size ^2^	
Carriers, n (%)	26 (16%)
Non-carriers, n (%)	139 (84%)
Apo(a) phenotype ^2^	
Single expressed isoform, n (%)	48 (29%)
Two expressed isoforms, n (%)	117 (71%)
Apo(a) isoform dominance pattern ^3^	
Co-dominating, n (%)	73 (63%)
Smaller-dominating, n (%)	39 (33%)
Larger-dominating, n (%)	5 (4%)

Data are shown as the median (IQR) number (%), or, for changes, mean ± SD. ^1^: *p* < 0.0001 for unit (mg/dL) and percent (%) changes between the two diets [17]. ^2^: Based on data from participants with at least one visible apo(a) protein band on Western blot. One participant with no visible apo(a) protein band was excluded; thus, data were obtained from 165 participants. ^3^: Based on the data of 117 participants with two expressed apo(a) isoforms. Abbreviations: Apo(a)—apolipoprotein(a); DASH—Dietary Approaches to Stop Hypertension; Lp(a)—lipoprotein(a).

**Table 2:** The relationship between changes in lipoprotein(a) level and carrier status of a small (≤22 kringles)-size apolipoprotein(a).

Lp(a) Level	Carriers (n = 26)	Non-Carriers (n = 139)	*p*-Value *
Average American Diet (mg/dL)	100 (47–120)	38 (21–68)	<0.0001
DASH-type diet (mg/dL)	111 (53–129)	52 (26–85)	<0.0001
Unit (mg/dL) change	15 ± 14	11 ± 11	0.114
Percent (%) change	21 ± 20	25 ± 26	0.518

Data shown as median (IQR) for Lp(a) level and mean ± SD for changes. *: *p*-values are for differences between carriers vs. non-carriers. Abbreviations: DASH—Dietary Approaches to Stop Hypertension; Lp(a)—lipoprotein(a).

**Table 3:** Lipoprotein(a) level and apolipoprotein(a) characteristics across tertiles of combined kringle repeats.

Variables	Tertiles of Combined Apo(a) Kringles *			
	Tertile 1 (<54 K) (n = 34)	Tertile 2 (>54 K and <61 K) (n = 43)	Tertile 3 (>61 K) (n = 40)	*p*-Value ^#^
Lp(a) level				
Average American Diet (mg/dL)	98 (80–113)	46 (33–77)	29 (18–50)	<0.0001
DASH-type diet (mg/dL)	111 (88–129)	64 (41–91)	39 (20–62)	<0.0001
Unit (mg/dL) change	15 ± 14	14 ± 10	8 ± 9	0.024/0.006
Percent (%) change	17 ± 16	28 ± 19	24 ± 31	0.104/0.298
Apo(a) dominance pattern				
Co-dominating, n (%)	23 (68)	31 (72)	19 (48)	0.060
Smaller-dominating, n (%)	8 (23)	10 (23)	21 (52)	0.009
Larger-dominating, n (%)	3 (9)	2 (5)	0 (0)	0.191
Prevalence of a small (<22 kringles) size apo(a)				
Carrier, n (%)	19 (56)	3 (7)	0 (0)	<0.001
Non-carrier, n (%)	15 (44)	40 (93)	40 (100)	<0.001

Data are expressed as median (IQR), number (%), or, for changes, mean ± SD. *: Determined only for two expressed apolipoprotein(a) isoforms (n = 117). ^#^: *p*-value is for differences between the tertile groups/*p*-value for trend across tertile groups. Abbreviations: Apo(a)—apolipoprotein(a); DASH—Dietary Approaches to Stop Hypertension; Lp(a)—lipoprotein(a).

**Table 4:** Regression analysis for Lp(a) changes without or with covariates in all participants.

Variables	Level Change (mg/dL)				Relative Change (%)			
	Before Adjustment		After Adjustment		Before Adjustment		After Adjustment	
	β	*p*-Value	β	*p*-Value	β	*p*-Value	β	*p*-Value
Age	−0.011	0.898	−0.005	0.946	−0.017	0.930	0.012	0.954
Sex	0.630	0.746	−0.679	0.722	−3.334	0.443	−1.629	0.722
AAD Lp(a) level	0.113	<0.001	0.098	<0.001	−0.126	0.014	−0.144	0.032
Apo(a) phenotype (single or two)	2.698	0.171	−1.510	0.871	−2.592	0.560	0.412	0.986
Size of small apo(a) isoform	−0.832	<0.001	−0.354	0.404	0.472	0.355	−0.344	0.743
Combined kringle repeats	−0.010	0.872	0.040	0.886	−0.017	0.901	−0.013	0.985

Data from all participants were used for regression analysis (n = 165). The regression model was fit via mixed-effects models with a random intercept for each family. Adjustments were made for all covariates, including age, sex, AAD Lp(a) level, number of expressed apo(a) isoforms, size of the small apo(a) isoform, and combined kringle repeats from two isoforms. For continuous covariates, the beta coefficient corresponds to 1 unit increase in covariate (e.g., age from 50 to 51 years). Abbreviations: AAD—Average American Diet; Apo(a)—apolipoprotein(a).

## Data Availability

The original contributions presented in this study are included in the article/Supplementary Materials. Further inquiries can be directed to the corresponding author Enkhmaa Byambaa (ebyambaa@ucdavis.edu).

## Supplementary Materials

The following supporting information can be downloaded at https://www.mdpi.com/article/10.3390/nu17030426/s1, Table S1. Changes in other measured biomarkers from the AAD diet to the DASH-type diet. *: A log-transformation was used to test significance for non-normally distributed variables. Abbreviations: Ln—log transformation; PAI-1—plasminogen activator inhibitor type 1. Table S2: The relationship between changes in lipoprotein(a) level and apolipoprotein(a) dominance patterns. Data shown as median (IQR), except for changes shown as mean ± SD. *: No significant differences between apo(a) isoform dominance pattern groups for all variables (*p* > 0.05). Abbreviations: Apo(a)—apolipoprotein(a); DASH—Dietary Approaches to Stop Hypertension; Lp(a)—lipoprotein(a). Table S3: The relationship between LDL-C change and carrier status of a small-size apo(a) (≤22 kringle repeats). Both unit and percent changes are expressed as mean ± SD. Abbreviations: Apo(a)—apolipoprotein(a); AAD—Average American Diet; DASH—Dietary Approaches to Stop Hypertension Diet. Table S4: The relationship between changes in LDL-C level and apo(a) dominance patterns. Data are shown as mean ± SD. *: Assessed for the 117 participants with two expressed apo(a) isoforms. #: *p* < 0.05 for differences across dominance pattern subgroups (co- vs. smaller- vs. larger- dominating). Abbreviations: AAD—Average American Diet; DASH—Dietary Approaches to Stop Hypertension Diet. Table S5: LDL-C and apo(a) characteristics across tertiles of combined apo(a) kringle repeats. Data are shown as mean ± SD. *: Assessed for the 117 participants with two expressed apo(a) isoforms. #: *p*-value is for differences between the tertile groups/trend across tertiles for changes, respectively. Abbreviations: Apo(a)—apolipoprotein(a); AAD—Average American Diet; DASH—Dietary Approaches to Stop Hypertension Diet; K—kringles; T—tertile. Figure S1: Distributions of Lp(a) levels during the consumption of the average American diet and the DASH-type diet in African Americans. The chart illustrates the distribution of Lp(a) levels using cutoffs recommended by clinical guidelines in the same individuals undergoing two different dietary interventions in the GET-READI cohort (n = 166). During the consumption of the Average American Diet (AAD) containing 16% dietary saturated fat (SFA) as daily energy requirements (E%), only 44% (n = 73) of participants had an Lp(a) level of >50 mg/dL, representing the high-risk category (the left panel). This proportion rose to 56% (n = 93) when participants consumed the DASH-type diet with 6% SFA as E% (the right panel). Conversely, the proportion of individuals who had an Lp(a) level of ≤30 mg/dL, representing the low-risk category, was reduced from 34% (n = 57) during the AAD intervention to 25% (n = 42) during the DASH-type diet intervention. These observations suggest that dietary SFA reduction induced increases in Lp(a) levels could be substantial in some individuals, resulting in an unfavorable shift in their Lp(a)-attributable risk category.

## Abbreviations

The following abbreviations are used in this manuscript:

AAD	Average American Diet.
Apo(a)	Apolipoprotein(a).
ASL	Allele-specific apo(a) level.
CKD	Chronic kidney disease.
CVD	Cardiovascular disease.
DASH	Dietary Approaches to Stop Hypertension.
K	Kringles.
Lp(a)	Lipoprotein(a).
SFA	Saturated fatty acid.
