# Supplementary material for: Lipoprotein(a) Response to Dietary Saturated Fat Reduction: Relationship to Apolipoprotein(a) Size Polymorphism in African Americans
Source: Nutrients. 2025 Jan 24;17(3):426. doi: 10.3390/nu17030426 (PMC11820444; doi:10.3390/nu17030426)
Supplement: Supplementary file 1 [file nutrients-17-00426-s001.zip › nutrients-3438099-supplementary.pdf]

SUPPLEMENTARY MATERIALS

Article

Lipoprotein(a) Response to Dietary Saturated Fat Reduction: Relationship to Apolipoprotein(a) Size Polymorphism in African Americans

Hayley G. Law [1], Munkhtuya Myagmarsuren [1], Heejung Bang [2], Wei Zhang [1], Michael Lefevre [3], Lars Berglund [1] and Byambaa Enkhmaa [1,*]

[1] Department of Internal Medicine, School of Medicine, University of California Davis, One Shields Avenue, Davis, CA 95616, USA; haglaw@ucdavis.edu; mmyagmarsuren@ucdavis.edu; wzhang@ucdavis.edu; lberglund@ucdavis.edu; ebyambaa@ucdavis.edu

[2] Department of Public Health Sciences, School of Medicine, University of California Davis, One Shields Avenue, Davis, CA 95616, USA; hbang@ucdavis.edu

[3] Department of Nutrition, Utah State University, Old Main Hill, Logan, UT 84322, USA; michael.lefevre@usu.edu

* Correspondence: ebyambaa@ucdavis.edu; Tel.: +1 (530) 754-7253

Nutrients **2025**, *17*, 426

Table S1. Changes in other measured biomarkers from the AAD diet to the DASH-type diet

Variable	Mean ± SD	*P*-value
C-Reactive Protein * [ln(mg/dL)]	-0.11 ± 0.67	0.038
PAI-1 * [ln(ng/dL)]	-0.09 ± 0.034	0.008
Asymmetric dimethylarginine (ADMA)	-0.02 ± 0.16	0.110
Parathyroid hormone (pg/mL)	-0.48 ± 11.1	0.580
Angiotensin converting enzyme (ACE) (pg/mL)	0.84 ± 7.9	0.029
Renin (pg/mL)	1.57 ± 3.07	0.0001
Angiotensin-II	1.2 ± 5.8	0.015

*: A log transformation was used to test significance for non-normally distributed variables.

Abbreviations: . Ln—log transformation; PAI-1—plasminogen activator inhibitor type 1.

Table S2. The relationship between changes in lipoprotein(a) level and apolipoprotein(a) dominance patterns

**Lp(a) level**	**Apo(a) dominance pattern ***		
	**Co-dominating (n=73)**	**Smaller-dominating (n=39)**	**Larger-dominating (n=5)**
Average American diet (mg/dL)	58 (31-97)	46 (25-78)	55 (40-56)
DASH-type Diet (mg/dL)	68 (38-111)	60 (34-89)	77 (50-83)
Unit (mg/dL) change	13 ± 12	10 ± 10	20 ± 15
Percent (%) change	25 ± 26	19 ± 19	29 ± 19

Data shown as median (IQR), except for changes shown as mean ± SD.

*: No significant differences between apo(a) isoform dominance pattern groups for all variables (p>0.05).

Abbreviations: Apo(a)—apolipoprotein(a); DASH—Dietary Approaches to Stop Hypertension; Lp(a)—lipoprotein(a)

Table S3: The relationship between LDL-C change and carrier status of a small (≤22 kringle repeats) size apo(a)

**LDL-C level**	**Carriers (n=26)**	**Non-carriers (n=139)**	***p*-value**
End of AAD (mg/dL)	114 ± 21	116 ± 29	0.678
End of DASH-type (mg/dL)	101 ± 17	104 ± 27	0.467
Unit (mg/dL) change	-13 ± 12	-12 ± 16	0.739
Percent (%) change	-11 ± 9	-10 ± 13	0.708

Both unit and percent changes are expressed as mean ± SD.

Abbreviations: Apo(a)—apolipoprotein(a); AAD—Average American Diet; DASH—Dietary Approaches to Stop Hypertension Diet

Table S4. The relationship between changes in LDL-C level and apo(a) dominance patterns

LDL-C level	Apo(a) isoform dominance patterns *		
	**Co-dominating (n=73)**	**Smaller-dominating (n=39)**	**Larger-dominating (n=5)**
End of AAD (mg/dL) #	118 ± 30	108 ± 20	138 ± 17
End of DASH-type diet (mg/dL) #	106 ± 25	93 ± 17	114 ± 10
Unit (mg/dL) change	-12 ± 16	-14 ± 12	-23 ± 17
Percent (%) change	-9 ± 12	-13 ± 11	-16 ± 12

Data are shown as mean ± SD.

*: Assessed for the 117 participants with two expressed apo(a) isoforms.

#: $p<0.05$ for differences across dominance pattern subgroups (co- vs smaller- vs larger- dominating).

Abbreviations: AAD—Average American Diet; DASH—Dietary Approaches to Stop Hypertension Diet

Table S5. LDL-C and apo(a) characteristics across tertiles of combined apo(a) kringle repeats

LDL-C level	Tertiles of combined apo(a) kringles *			
	**T1 (<54K) (n=34)**	**T2 (≥54 and <61K) (n=43)**	**T3 (≥61K) (n=40)**	***p*-value #**
End of AAD (mg/dL)	113 ± 22	119 ± 27	114 ± 33	0.613/0.478
End of DASH-type (mg/dL)	100 ± 18	106 ± 23	100 ± 26	0.352/0.433
Unit (mg/dL) change	-13 ± 13	-12 ± 13	-14 ± 19	0.804/0.922
Percent (%) change	-11 ± 11	-10 ± 10	-11 ± 14	0.837/0.988

Data are shown as mean ± SD.

*: Assessed for the 117 participants with two expressed apo(a) isoforms.

#: *p*-value is for differences between the tertile groups/trend across tertiles for changes, respectively.

Abbreviations: Apo(a)—apolipoprotein(a); AAD—Average American Diet; DASH—Dietary Approaches to Stop Hypertension Diet; K—kringles; T—tertile

Figure S1. Distributions of Lp(a) levels during the consumption of the average American diet and the DASH-type diet in African Americans

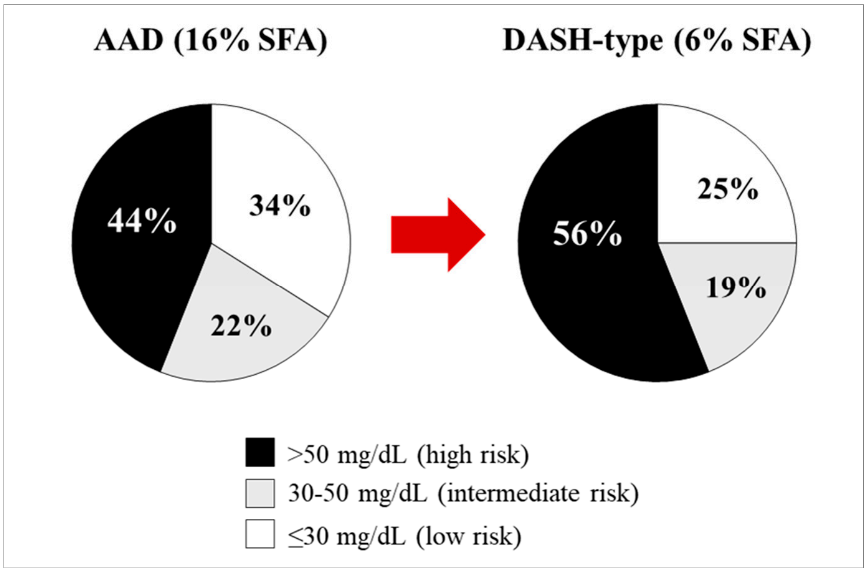

The chart illustrates the distribution of Lp(a) levels using cutoffs recommended by clinical guidelines in the same individuals undergoing two different dietary interventions in the GET-READI cohort (n=166). During the consumption of the Average American diet (AAD) containing 16% dietary saturated fat (SFA) as daily energy requirements (E%), only 44% (n=73) of participants had an Lp(a) level of >50 mg/dL, representing the high-risk category (left panel). This proportion rose to 56% (n=93) when participants consumed the DASH-type diet with 6% SFA as E% (right panel). Conversely, the proportion of individuals who had an Lp(a) level of ≤30 mg/dL, representing the low-risk category, was reduced from 34% (n=57) during the AAD intervention to 25% (n=42) during the DASH-type diet intervention. These observations suggest that dietary SFA reduction induced increases in Lp(a) levels could be substantial in some individuals, resulting in an unfavorable shift in their Lp(a)-attributable risk category. Abbreviations: Lp(a)—Lipoprotein(a);AAD—Average American Diet; DASH—Dietary Approaches to Stop Hypertension Diet.
